# Dengue vector control in high-income, city settings: A scoping review of approaches and methods

**DOI:** 10.1371/journal.pntd.0012081

**Published:** 2024-04-17

**Authors:** Anna Durrance-Bagale, Nirel Hoe, Jane Lai, Jonathan Wee Kent Liew, Hannah Clapham, Natasha Howard

**Affiliations:** 1 Saw Swee Hock School of Public Health, National University of Singapore and National University Health System, Singapore, Singapore; 2 London School of Hygiene & Tropical Medicine, Department of Global Health & Development, London, United Kingdom; 3 Environmental Health Institute, National Environment Agency, Singapore, Singapore; National Center for Atmospheric Research, UNITED STATES

## Abstract

**Background:**

Dengue virus (DENV) is endemic to many parts of the world and has serious health and socioeconomic effects even in high-income countries, especially with rapid changes in the climate globally. We explored the literature on dengue vector control methods used in high-income, city settings and associations with dengue incidence, dengue prevalence, or mosquito vector densities.

**Methods:**

Studies of any design or year were included if they reported effects on human DENV infection or *Aedes* vector indices of dengue-specific vector control interventions in high-income, city settings.

**Results:**

Of 24 eligible sources, most reported research in the United States (n = 8) or Australia (n = 5). Biocontrol (n = 12) and chemical control (n = 13) were the most frequently discussed vector control methods. Only 6 sources reported data on the effectiveness of a given method in reducing human DENV incidence or prevalence, 2 described effects of larval and adult control on *Aedes* DENV positivity, 20 reported effectiveness in reducing vector density, using insecticide, larvicide, source reduction, auto-dissemination of pyriproxyfen and *Wolbachia*, and only 1 described effects on human-vector contact.

**Conclusions:**

As most studies reported reductions in vector densities, rather than any effects on human DENV incidence or prevalence, we can draw no clear conclusions on which interventions might be most effective in reducing dengue in high-income, city areas. More research is needed linking evidence on the effects of different DENV vector control methods with dengue incidence/prevalence or mosquito vector densities in high-income, city settings as this is likely to differ from low-income settings. This is a significant evidence gap as climate changes increase the global reach of DENV. The importance of community involvement was clear in several studies, although it is impossible to tease out the relative contributions of this from other control methods used.

## Author summary

Dengue virus is present in many parts of the world and has serious health and socioeconomic effects even in high-income countries, especially with rapid changes in the climate globally. In this study, we explored the literature on dengue vector control methods used in high-income, city settings and associations with number of human dengue infections, and the density of mosquitoes. We found 24 papers with relevant results. Most of these described studies in the United States or Australia. Most were about various forms of biological or chemical control of the mosquitoes. Few papers discussed effects on human dengue infection. We were unable to draw any clear conclusions on how effective mosquito control methods were as we could identify little research on this subject in this specific setting. More research is needed on this topic, particularly as climate change will make more areas of the world vulnerable to dengue infection.

## Introduction

Dengue, a vector-borne viral disease transmitted through the bite of an infected female *Aedes* mosquito, is estimated to cause 390 million infections annually, of which 96 million manifest clinically and cause a major healthcare burden [1,2]. Dengue virus (DENV) is endemic in over 100 countries globally, with Asia representing around 70% of the global burden [1] of 3.9 billion people at risk [2,3]. Although most infections cause mild symptoms, DENV can cause severe disease and fatality [4]. Dengue also has serious socioeconomic effects. For example, in Singapore, a hyperendemic high-income island nation, the economic impact of dengue in direct medical costs and lost productivity was estimated at US$1 billion annually for 2000–2009 [5].

Given infection risks, the lack of sufficiently effective vaccines or dedicated treatment, and high socioeconomic burden, effective and sustainable vector control is crucial to reduce dengue transmission. *Aedes aegypti*, the primary vector globally, breeds in both natural and artificial habitats, including used tyres, containers, and storm drains, so is frequent in urban areas. Eggs remain dormant and viable for several months if kept dry and hatch when in contact with water, underlining the importance of emptying water containers frequently. *Aedes albopictus*, a secondary dengue vector in Asia, is also commonly found in urban areas and has been detected in more than 25 countries in Europe and 32 US states [1].

The World Health Organization (WHO) lists four main dengue vector control approaches, ideally as part of integrated vector management: (i) chemical control (i.e. the use of insecticides to kill or reduce mosquito populations, including space spraying, residual spraying, larviciding, autodissemination); (ii) biological control (i.e. the use of natural enemies to control mosquito populations, including fish, copepods, *Bacillus thuringiensis israelensis* [Bti], *Wolbachia*); (iii) source reduction (i.e. eliminating or reducing mosquito breeding sites, including by removing or covering water storage containers, proper disposal of solid waste, cleaning gutters and drains, along with community mobilisation and advocacy); and (iv) personal or barrier protection (i.e. using window screens, mosquito netting, repellents, or protective clothing to avoid mosquito bites) [6]. We focused on vector control methods in high-income, city settings because a preliminary review suggested a broader range of methods and implementation intensities could remain cost-effective in densely populated higher-income urban settings. Additionally, although dengue has historically been a disease affecting resource-poor settings, climate change is increasing its global reach [7].

We aimed to explore the literature on dengue vector control methods used in high-income city settings and any associations identified with dengue incidence, dengue prevalence, or mosquito vector densities. Our objectives were to: (i) summarise the scope (i.e. extent, nature, distribution) of the existing literature; (ii) synthesise main findings and lessons on the effects of vector control methods used in high-income settings on dengue incidence, dengue prevalence, and mosquito vector densities; and (iii) identify any significant gaps in the literature that warrant further research.

## Methods

### Study design

We conducted a scoping review using Arksey and O’Malley’s multi-stage scoping method, informed by Levac *et al*’s 2010 revisions and Khalil *et al*’s 2016 refinements [8–11]. Table 1 shows our study definitions. We chose the World Bank definition of cities as ‘having a population of at least 50,000 in contiguous dense grid cells with a density of over 1,500 inhabitants per km^2^’ rather than the broader ‘urban’ terminology to facilitate international comparability with our own Singaporean context [12].

**Table 1:** Study definitions.

City	A population of at least 50,000 in contiguous dense grid cells with a density of over 1,500 inhabitants per km^2^ [12].
Dengue fever	Dengue fever is the most prevalent viral infection transmitted by *Aedes* mosquitoes, with more than 3.9 billion people in over 129 countries at risk of contracting dengue, and an estimated 96 million symptomatic cases and 40,000 deaths annually [13].
High-income	For the 2022 fiscal year, high-income economies are defined by the World Bank as those with a gross national annual income per capita of at least US$12,696 [14].
Urban	Includes: (i) cities, which have a population of at least 50,000 in contiguous dense grid cells with a density of over 1,500 inhabitants per km^2^; and (ii) towns/semi-dense areas, which have a population of at least 5,000 inhabitants in contiguous grid cells and density of at least 300 inhabitants per km^2^ [12].
Vector	Living organisms that can transmit infectious pathogens between humans, or from animals to humans [13].
Vector control	Vector control aims to limit the transmission of pathogens by reducing or eliminating human contact with the vector [15].

### Stage 1. Defining research questions

Our research questions were: (i) ‘what is the scope and main findings of the existing literature on dengue vector control in high-income, city settings?’ and (ii) ‘which of these vector control methods appear to be associated with the greatest reductions in dengue incidence, prevalence or vector densities?’

### Stage 2. Identifying potentially relevant sources

First, we searched eight electronic databases and websites systematically using terms and related terminology for ‘city’ AND ‘dengue’ AND ‘vector control’ adapted to subject headings for each database (i.e. EMBASE, Medline, Web of Science, Global Index Medicus, Cochrane Central Register of Controlled Trials; see Table 2 for example Medline search). Second, we searched selected websites purposively (i.e. clinicaltrials.gov, ISRCTN registry, WHO International Clinical Trials Registry Platform).

**Table 2:** Medline search syntax.

#	Query	Results from 30 Apr 2022
1	exp Dengue/	14,971
2	Dengue Virus/	10,275
3	(dengue or break-bone or breakbone).mp.	26,765
4	1 or 2 or 3	26,765
5	Aedes/	16,885
6	exp Mosquito Vectors/	5,138
7	(aedes or mosquito* or vector*).mp.	353,338
8	5 or 6 or 7	353,338
9	4 and 8	10,752
10	(urban or city or cities or town or towns or metropolitan* or suburb*).mp.	416,239
11	Urban Population/	62,221
12	10 or 11	416,239
13	9 and 12	1,814

### Stage 3. Selecting eligible sources

Table 3 shows eligibility criteria, established iteratively based on our research questions. We restricted context to high-income, city settings and topics to dengue vector control as defined in Table 1. We restricted outcomes to case incidence/prevalence, adult mosquito density, entomological inoculation or landing rate, *Aedes* DENV-positivity rate, or adverse/unintended effects, and restricted source types to primary research, but did not restrict time-period, study design, participants, or publication language if we could access an English abstract (Table 2).

**Table 3:** Eligibility criteria.

Criteria	Included	Excluded
Context	• High-income urban settings (e.g. towns, cities, suburbs)	• Other settings (e.g. low-income, lower middle-income, upper middle-income) • Rural and peri-urban settings
Topic	• Dengue-specific vector control intervention (e.g. larval source management, fogging, spatial repellents, window screens)	• Studies unrelated to dengue-specific mosquito control (e.g. malaria vector control, *Culicine* vector control)
Outcomes	• Effects on human DENV infection or *Aedes sp*. vector indices (e.g. *Aedes* DENV-positivity rate, adult mosquito density, landing rate). • Adverse events/unintended effects, e.g.: (i) toxicity to humans/animals; (ii) environmental impacts, such as changes to biodiversity; (iii) changes to levels of phenotypic/molecular insecticide resistance; (iv) changes in mosquito species composition (e.g. species replacement or behaviour that reduces vector control intervention efficacy such as exophily, exophagy, biting time)	• Other outcomes
Source type	• Primary research articles • Commentaries/editorials that include primary research • Conference abstracts that include primary research	• Secondary/tertiary sources (e.g. review articles, meta-analyses, textbooks, dictionaries) • Audio/video reports • Conference abstracts covering the same material as an available publication • Social media, blogs, media articles • Guidance/legal documents
Time-period	• All	• NA
Language	• All for which an English abstract is available	• Sources for which no English abstract is accessible
Study design	• Any	• NA
Participants	• Any	• NA

**Fig 1:**
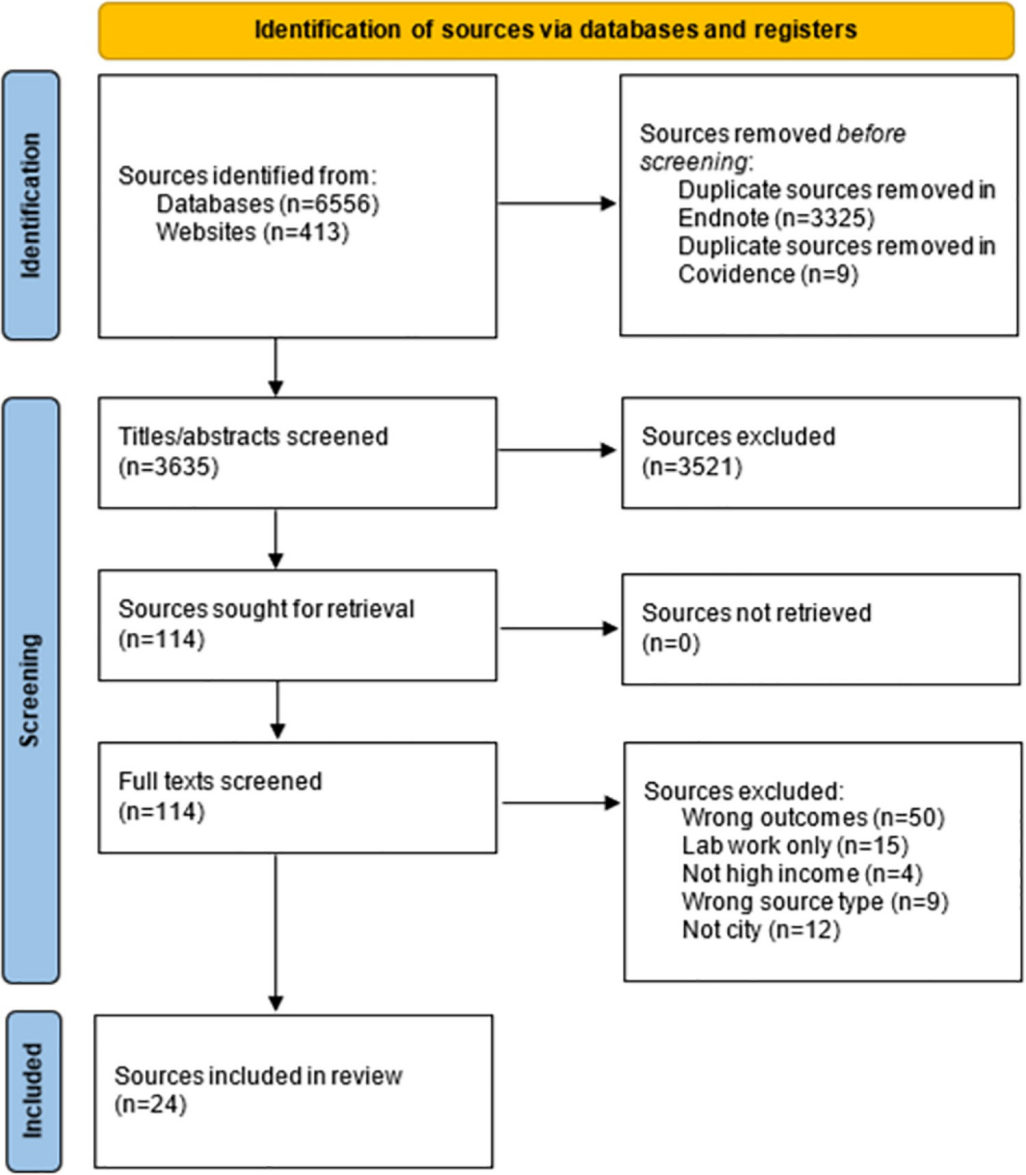
PRISMA flow diagram.

After downloading potential sources from databases or websites and de-duplicating in EndNote reference manager, we first screened titles and abstracts and then full texts against eligibility criteria using Covidence software to remove ineligible documents. Finally, we purposively searched reference lists of all included sources to include additional eligible sources. This provided our total number of included documents (Fig 1).

### Stage 4. Extracting data

We extracted data to an Excel sheet using the following headings: source identifiers, i.e. publication year, lead author; source type (e.g. article, conference abstract, report); source characteristics, i.e. country, study design; and findings on effectiveness of dengue vector control methods.

### Stage 5. Synthesising and reporting results

First, we quantified the scope of our sources by extent (i.e. publication year, type), distribution (i.e. by country, publication language), and nature (i.e. study design, topics, outcomes). Second, we synthesised findings data descriptively in alignment with our research objectives, as they were too heterogenous to allow quantitative analyses, and considered implications for policy, practice, and further research.

## Results

### Scope of the literature

Fig 1 presents the PRISMA flow diagram for the 24 eligible sources included of 6,969 identified in our April 2022 searches.

**Fig 2:**
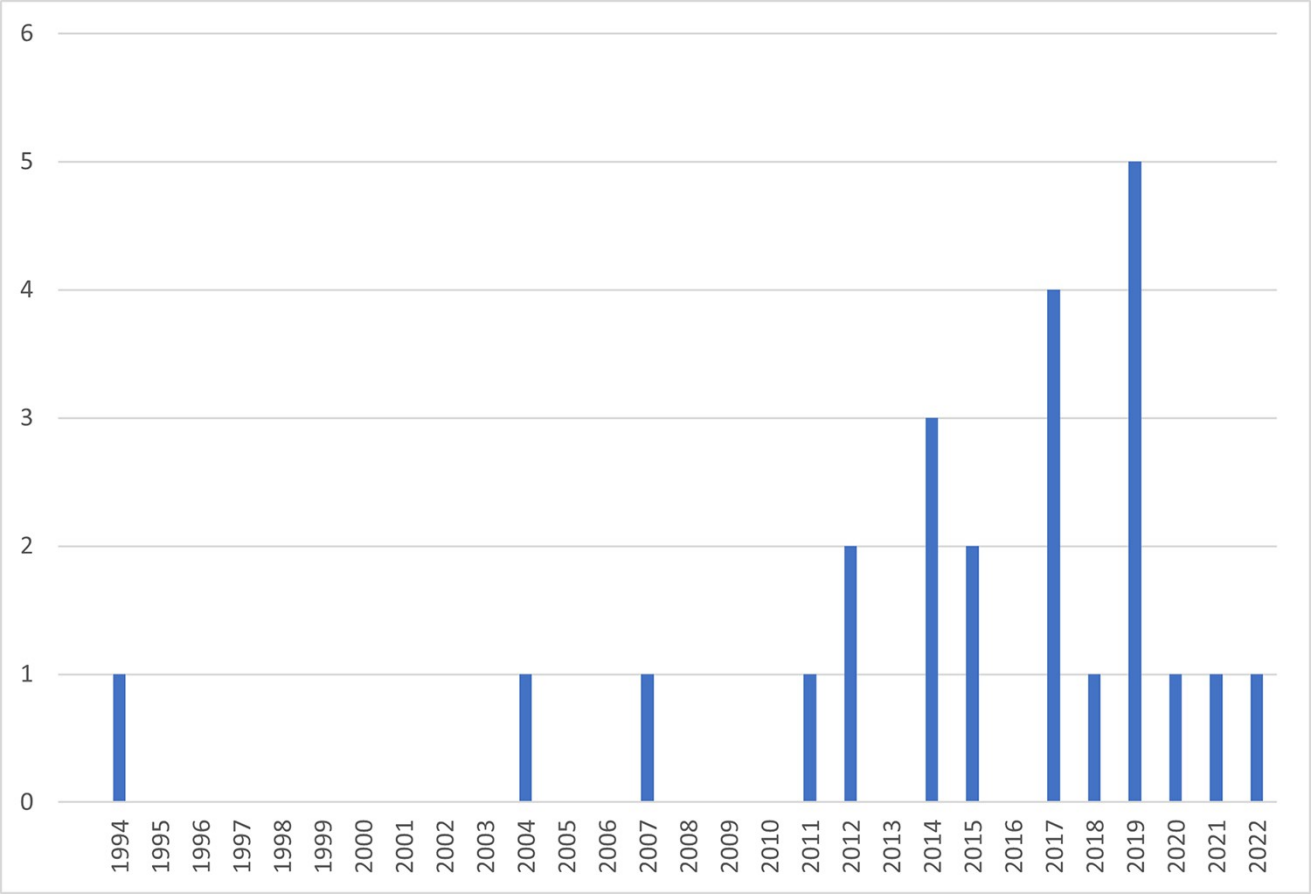
Sources by publication year.

Fig 2 shows the number of sources by publication year, indicating that published research on this subject appears to have increased somewhat after 2014. Twenty-three (96%) sources were peer-reviewed journal articles, with one abstract. Sources reported research in two countries in the Americas (US and Uruguay; n = 9), one in Oceania (Australia; n = 5), four in Europe (Spain, Germany, Italy, and France; n = 6), and one in Asia (Taiwan; n = 4). All sources were single-country. The United States (n = 8) and Australia (n = 5) dominated sources while Spain, Uruguay and France were only included in 1 source each.

All were published in English, drawing from public health, epidemiology, and economics disciplines. Study designs included controlled (n = 11), uncontrolled (n = 6), randomised controlled (n = 1), cluster-randomised (n = 1), and cluster randomised stepped-wedge (n = 1) trials; and a time series study (n = 1). Three studies evaluated a programme/strategy.

Table 4 shows the most common vector control methods were biocontrol, using *Bacillus thuringiensis israelensis* (Bti; n = 9), *Wolbachia* (n = 3), and chemical control, as either methoprene (n = 1), insecticide (n = 8), autodissemination (n = 2) or larvicide (n = 2), and environmental source reduction (n = 2).

**Table 4:** Sources by lead author and publication year.

First author (year)	Country	Vector control methods	Study design	Outcome measure	Effects on human DENV incidence/ prevalence	Effects on *Aedes* DENV-positivity	Effects on vector density	Effects on vector-human contact	Cost-effectiveness
Abramides (2011)	Spain	Insecticide (alfacipermetrin); larvicide (Bti; diflubenzuron)	Controlled trial	Vector abundance			X		
Barrera (2019)	USA—Puerto Rico	Larvicide (Bti); source reduction	Cluster-randomised, stepped-wedge	*Aedes* DENV positivity; vector density		X	X		
Basso (2015)	Uruguay	Source reduction	Cluster-randomised trial	Vector density			X		X
Becker (2017)	Germany	Larvicide (Bti)	Programme/ strategy evaluation	Community participation; vector density			X		
Becker (2022)	Germany	Larvicide (Bti)	Programme/ strategy evaluation	Community participation; vector density			X		X
Caputo (2012)	Italy	Auto-dissemination (pyriproxyfen)	Controlled trial	Pupal mortality			X		
Chen (1994)	Taiwan	Larvicide (Temephos)	Uncontrolled trial	Larval mortality	X		X		
Chen (2020)	Taiwan	Auto-dissemination (pyriproxyfen)	Controlled trial	Larval mortality			X		
Darbro (2017)	Australia	Insecticide (metofluthrin)	Controlled trial	Knockdown rate				X	
Farajollahi (2012)	USA	Insecticide (DUET)	Controlled trial	Vector density			X		
Garcia-Luna (2019)	USA	Larvicide (Bti)	Controlled trial	Vector density			X		
Harris (2021)	USA—Puerto Rico	Larvicide (Bti)	Controlled trial	Vector density			X		
Mains (2019)	USA	*Wolbachia*	Controlled trial	Vector density			X		
Marini (2015)	Italy	Insecticide (Etox; Microsin)	Uncontrolled trial	Vector density			X		
O’Neill (2018)	Australia	*Wolbachia*	Time series study (intervention)	Vector density; human DENV incidence/prevalence	X				
Pai (2014)	Taiwan	Insecticide (permethrin/cypermethrin)	Uncontrolled trial	Vector density			X		
Pruszynski (2017)	USA	Larvicide (Bti)	Controlled trial	Vector density			X		
Ritchie (2004)	Australia	Insecticide (S-methoprene/lambda-cyhalothrin)	Uncontrolled trial	Vector density; human DENV incidence/prevalence; *Aedes* DENV positivity	X	X	X		
Ryan (2019)	Australia	*Wolbachia*	Uncontrolled trial	Human DENV transmission	X				
Sun (2014)	USA	Larvicide (Bti)	Controlled trial	Vector density			X		X
Teng (2007)	Taiwan	Insecticide (deltamethrin; permethrin); source reduction	Programme evaluation	Vector density; human DENV incidence/prevalence	X		X		
Thuilliez (2019)	France—Réunion	Source reduction	Randomised, controlled trial	Vector density			X		
Vazquez-Prokopec (2017)	Australia	Insecticide (lambda-cyhalothrin)	Uncontrolled trial	Human DENV incidence/prevalence	X				
Williams (2014)	USA	Larvicide (Bti)	Controlled trial	Larval mortality			X		

### Effectiveness in reducing human DENV incidence or prevalence

Table 4 shows 6 sources reported effects of interventions (i.e. indoor residual spraying, use of larvicide or adulticide, or release of *Wolbachia*-infected mosquitoes) on human DENV incidence or prevalence [16–21].

O’Neill et al conducted a time-series study of large-scale *Wolbachia* introduction into an *Ae*. *aegypti* population over a 28-month period, and found it was associated with a reduced incidence of locally acquired dengue cases in a population of just under 200,000 residents in Townsville, Australia (i.e. 4 versus 54 cases in the preceding 44-month period) [17]. Community members participated in releasing *Wolbachia*-infected *Ae*. *aegypti* mosquitoes, which was cost-effective and allowed targeted deployment to ensure sufficient coverage in each area [17].

Ryan et al conducted an uncontrolled trial of *Wolbachia* in Australia showing a 96% (95% CI 84–99%) reduction in dengue incidence after establishment of *Wolbachia-Ae*. *aegypti* in the population, suggesting near-elimination of locally acquired dengue in *Wolbachia*-treated communities [19]. Part of this ‘*Wolbachia* Warriors’ programme involved students, teachers and parents rearing and releasing mosquitoes at home, which also increased awareness of mosquito biology [19].

Ritchie et al conducted an uncontrolled intervention trial in suburban Cairns, Australia showing that combined larval and adult control using containers treated with S-methoprene or lambda-cyhalothrin reduced dengue incidence by almost 50% from 7 to 4 cases daily over a 3-week period, though further details were not provided [18].

Two intervention trials reported effects of larviciding and adulticiding on dengue incidence in Taiwan [16,20]. Chen et al found that a campaign to reduce breeding sites, using Temephos larvicide or larvivorous fish, reduced dengue fever incidence by 98% from 1988 (time of the intervention) to 1994, with no cases detected in 1990 or 1993 [16]. Teng et al found that, when insecticide space spraying was combined with larval and adult mosquito surveys within 100 metres of a suspected dengue case, the number of cases decreased by 50% [20]. However, further detail on incidence calculations were not provided.

Vazquez-Prokopec et al conducted a modelling study based on data from a 2008–2009 dengue outbreak in Cairns (902 confirmed cases) and found that contact tracing combined with targeted indoor residual spraying of lambda-cyhalothrin insecticide in locations where people were potentially exposed could have reduced the likelihood of DENV transmission by 86–96% compared to unsprayed areas [21].

### Effectiveness in reducing Aedes DENV-positivity

Two sources described effects of larval or adult control on *Aedes* DENV positivity [18,22]. Using lambda-cyhalothrin, Ritchie et al successfully eliminated detectable virus in female *Ae*. *aegypti*: of six positive pools, one pool remained dengue-positive 4 weeks after control initiation, reducing to no virus detection in the next 6 weeks [18]. Barrera et al conducted a cluster-randomised, stepped-wedge study of larviciding (Bti) and source reduction during a 2016 outbreak in Puerto Rico. They found that larviciding an urban area of 23 km^2^ did not reduce numbers of DENV-positive pools. Of 12,081 mosquito pools sampled, only one was DENV-positive pre-intervention (October 2016-March 2017), and one was positive post-intervention (July 2017) [22].

### Effectiveness in reducing mosquito vector densities

Twenty sources reported effectiveness in reducing vector densities, using biocontrol [Bti (n = 9), *Wolbachia* (n = 1)], chemical control [insecticide (n = 4), methoprene (n = 1), autodissemination (n = 2), larvicide (n = 1)], and source reduction methods (n = 4).

Several sources only described combined interventions. For example, Abramides and colleagues examined the effectiveness of larviciding (diflubenzuron), Bti, adulticiding (alfacipermetrin), and source reduction through clearance of landfill sites in six neighbourhoods in Spain, using a quasi-experimental design, and demonstrated that areas with interventions had significantly fewer mosquito eggs than control areas: median number of eggs in intervention and control areas was 172 and 272, respectively in 2008, and 884 and 1668 eggs, respectively, in 2009 [23]. They also investigated citizens’ responses to source-reduction and door-to-door communication, showing 16% (95% CI 13–19%) more people agreed to house inspection in the second year [23].

Becker and colleagues three-arm intervention study of long-lasting door-to-door larviciding, sterile insect technique, and fizzy Bti tablets for *Ae*. *albopictus* control in three large urban areas Germany found larviciding was most effective with *Ae*. *albopictus* container index in one site reduced from 11% in 2019 to <1% in 2020 [24]. They found egg sterility was approximately 85% and 63% in intervention areas, compared with 15% in a control area [24]. Finally, community dissemination of fizzy Bti tablets (1 tablet/50L water bi-weekly) eliminated 100% of *Ae*. *albopictus* larvae, which was significantly different from controls, while 58% of residents who received a flyer describing ways to control mosquitoes effectively implemented control measures, were more aware of mosquito biology, and reported less mosquito nuisance [24,25].

### Biocontrol

#### Bti

Pruszynski and colleagues’ controlled trial of aerial applications of Bti to reduce female *Ae*. *aegypti* densities in Florida, USA., found weekly then biweekly Bti applications resulted in >55% mortality in larvae on application days, compared to <5% mortality in controls [26] and by Week 38 (the final application) numbers of female *Ae*. *aegypti* mosquitoes at treated sites were >50% lower than at control sites [26].

Two studies found that ground-based larvicide spray containing Bti decreased trapped *Ae*. *aegypti* abundance compared with control areas, in Texas (decreasing by 51% [12.83/trap/week to 5.56 trap/week] [27]) and Puerto Rico (Bti 500g/hectare applied weekly for 4 weeks and then fortnightly for 16 weeks was associated with 62% (p = 0.0001) and 28% (p<0.0001) reductions in adult female *Ae*. *aegypti* at treated sites compared with untreated [28]).

Sun and colleagues’ non-randomised controlled trial of the efficacy of motorised backpack applications of Bti and larviciding by hand to reduce *Ae*. *albopictus* density at six urban sites in New Jersey, USA [29], found larval mortality was 76% (standard error [SE] 68.2%) after backpack application and 92% (64.1% SE) after hand larviciding. Larval mortality was higher in hand-applied larviciding sites than in backpack sites (p<0.05), but significantly higher in backpack sites than in control sites (p<0.05) and the backpack method required 50-fold less labour than hand-applied larviciding [29].

Williams et al conducted an intervention study to investigate Bti’s larval mortality effects in US urban and suburban areas [30]. They found that cold aerosol fogger and misting machine applications of Bti killed an average 87% of *Ae*. *albopictus* larvae in residential areas, with efficacy maintained even in areas with trees and bushes [30].

#### Wolbachia

Mains et al investigated localised control of *Ae*. *aegypti* in Florida, after release of *Wolbachia*-infected male mosquitoes over 6 months in a controlled (treated versus untreated area) experimental study [31]. In the final 2 months of release, there was a significant 78% reduction in numbers of female *Ae*. *aegypti* in the central treated area compared with untreated areas [31].

### Chemical control

#### Methoprene

Larval control using S-methoprene or lambda-cyhalothrin successfully reduced numbers of female *Ae*. *aegypti*, concomitantly reducing dengue transmission in humans to zero [18].

Barrera et al found that larviciding a large urban area (23 km^**2**^) significantly reduced the mosquito population by 82% [22].

#### Insecticide

Marini et al compared pre- and post-treatment landing counts of *Ae*. *albopictus* after two insecticides (Etox and Microsin) and two application techniques (mist and stretcher sprayers) in densely populated areas of Italy and monitoring landing rates for 1 day before and 1, 7 and 14 days after each treatment [32]. Day 1 mosquito abundance decreased by 100% with both methods and insecticides and from 95% to 50% after 14 days (p<0.001). Stretcher sprayers were significantly more effective than mist sprayers for initial population reduction (78% versus 65%; p = 0.015) and 14-day reduction (60% versus 40%; p = 0.065) [32].

Farajollahi et al compared treated and untreated areas in urban New Jersey, USA in 2009–2011, and found that nocturnal application of ULV adulticide was effective at reducing *Ae*. *albopictus* abundance. A single application of 86.2 gm/hectare reduced abundance by 73%, which was significantly higher (p = 0.004) than an application of 42.7 gm/hectare (54%), but two applications at the lower concentration was the most effective (85% reduction, p = 0.003 compared with a single application at the full rate) [33].

Pai et al examined the performance of insecticidal aerosol cans in 20 randomly selected households in an urban residential area in Taiwan, finding that the indoor ovitrap index of permethrin-treated residences decreased from 60% before the control to 45% over the following 2 weeks. In residences treated with cypermethrin, the index decreased from 65% before control to 5% in week 1 and then 20% in weeks 2 and 3. Outdoor ovitrap indices also decreased: permethrin decreased from 90% to 65% in week 1 and then 80% in weeks 2 and 3. Cypermethrin decreased from 75% to 25% in week 1 and 5% in week 2 and 50% in week 3 [34]. In terms of acceptance of the insecticidal aerosol cans to be used around the home, only 20% of households regularly used insecticides. Of households contacted by local government during vector control campaigns, only 31% co-operated with indoor and outdoor administration of insecticides by government staff and 46% agreed to outdoor application only [34].

In a study evaluating an emergency control programme in response to dengue outbreaks in Taiwan, Teng and colleagues sprayed insecticide (deltamethrin and permethrin) using ULV or aerial space spray inside houses and ULV, aerial space spray or fogging on outdoor resting sites three times with a 7-day interval. They also used source reduction (reducing the availability of water containers in residences). These measures significantly reduced Breteau (51%) and larval (80%) indices, with no significant effects on the adult index, house index, container index, or by indoor, outdoor, or total water-filled containers per 100 premises examined. This resulted in a reduction in larvae of *Ae*. *albopictus* of 96% and in *Ae*. *aegypti* of 71% [20].

#### Auto-dissemination

Caputo et al conducted a controlled trial assessing feasibility of auto-dissemination in a cemetery and small park in Rome [35]. Adult females, exposed to resting sites contaminated with pyriproxyfen, spread this to larval habitats and prevented development and emergence of larvae. Each area included 10 dissemination sites contaminated with 5% pyriproxyfen powder, 10 sentinel sites and 10 covered, control sites. Each site contained 25 *Ae*. *albopictus* larvae, which were monitored for larval development and adult emergence. Significantly higher mortality was observed at the pupal stage in sentinel sites (50–70%) than in control sites (<2%), demonstrating that pyriproxyfen was transferred by the adult female mosquitoes and had a lethal effect on larvae [35].

Chen et al targeted cryptic habitats using *Ae*. *aegypti* mosquitoes as vehicles to transfer the pesticide pyriproxyfen to breeding sites inhibited larval emergence by 50% at 1 month after spraying in a field trial in Taiwan [36].

#### Larvicide

A breeding site reduction campaign in Taiwan, using both Temephos and larvivorous fish, found that *Aedes* larval density decreased by up to 91%, reflected in complete prevention of indigenous cases of human dengue [16]. Authors did not report mosquito species, although both *Ae*. *aegypti* and *Ae*. *albopictus* were reportedly present in study areas.

### Source elimination

Results from Teng et al [20] and Barrera et al [22] are reported in sections above. Thuilliez and colleagues reported results from a randomised controlled trial in Réunion aimed at measuring the efficacy of stagnant water source elimination in urban areas. They found that, 3 months post-intervention, properties visited by the public health agency (treated group) recorded more containers with larval or pupal mosquitoes than a control group who had not received any intervention [37]. The authors suggested that this might be a result of overcompensation, with treated households assuming that they were better protected by the habitat elimination, when in fact it should have been perceived as complementary to other methods of dealing with mosquitoes [37].

Basso et al used a cluster-randomised trial to investigate efficacy of an ecosystem management intervention in 20 randomly selected clusters in urban Salto in Uruguay [38], found the pupae per person index 1 month after the intervention increased in the intervention clusters by 2.7 times and in the control clusters by 8.7 times, although this difference was not statistically significant. Authors suggested the sample size was too small to detect a significant difference [38]. Residents were given informative flyers and tasked with collecting water-holding containers to be removed by the Ministry of Public Health [38]. Thirty-seven percent of households had collected containers, with the remaining households stating that they did not have any. Larger containers were modified by 75% of households so they could not hold water for mosquitoes to use (e.g. covered, punctured, turned upside down) [38].

#### Effectiveness in reducing human-vector contact

Only one source discussed reducing human-vector contact. Darbro and colleagues sought to decrease application time of indoor residual spraying in Queensland, Australia, using polyethylene netting impregnated with the pyrethroid metofluthrin in three rooms [39]. This effectively reduced *Ae*. *aegypti* landing and knockdown rates: at a 1-metre distance, a 10-minute exposure reduced indoor landing rates by up to 90% and increased knockdown rates by up to 90%. However, fewer effects were apparent at a 3-metre distance. Mosquitoes exposed to metofluthrin for >48 h had 100% and 90% mortality at 1 and 3 metres, respectively [39].

#### Cost-effectiveness

Only 3 sources included examination of cost-effectiveness as part of their study aims. A German programme involving door-to-door long-lasting larviciding, sterile insect technique, and community engagement in source reduction found that inspection and treatment cost between 6 and 8 euros per property, an estimated 9.5 euros per person/season, which made community collaboration cost-effective and sustainable [24].

A community campaign in Uruguay asked households to remove small water containers and alter larger containers so they could not be used to oviposit. The authors suggested that involving communities in this way could result in cost savings, and found that dengue vector density was reduced, although not statistically significantly [38].

In the USA, motorised backpack application of Bti to reduce *Ae*. *albopictus* densities in urban settings was much less labour-intensive and costly than hand-applied larviciding, i.e. US$160/hectare versus US$660/hectare, although efficacy was slightly lower using the backpack [29].

## Discussion

This review synthesises data on the technical and economic effectiveness of dengue vector control methods in high-income countries to reduce human dengue incidence or prevalence, *Aedes* DENV prevalence, and mosquito or larval densities. As effective dengue vector control using current tools is cost and labour intensive, requiring goodwill and active engagement from communities [40], it is imperative to ensure use of methods or combinations that work best in real world settings. However, we found limited effectiveness research in high-income settings, even though dengue is becoming a significant threat.

Most of the 24 sources included reported on research in the US and Australia, possibly because our search focused on high-income countries, though neither country is dengue endemic, indicating more evidence that publication is needed from endemic high-income countries. Most sources were published after 2014, suggesting increasing scientific interest in dengue vector control in high-income countries. However, most reported on reductions in vector density rather than human DENV incidence or prevalence, with some studies appearing to report effects on humans as an afterthought, rather than as a primary objective. This lack means we are unable to conclude from existing literature which intervention (or interventions) might be most effective or cost-effective in such settings.

In terms of which methods appear most promising in reducing human DENV incidence and prevalence, the population replacement approach using *Wolbachia*-infected mosquitoes was effective in reducing human DENV incidence, reportedly less labour-intensive than other approaches, and sufficiently acceptable in affected neighbourhoods, although results were only reported in two sources and how labour-intensiveness was measured and compared was not described [17,19].

It was similarly difficult to draw conclusions on the effectiveness of control methods at reducing *Aedes* DENV-positivity, as only two sources assessed this, although larviciding did appear moderately effective in one of these studies [18]. Only one study reported data on reduction in human-vector contact: although metofluthrin exposure was effective at decreasing knockdown and landing rates, mosquitoes had to be close to the emanator for optimal effects, making the feasibility of this control method questionable [39]. Few sources reported on cost-effectiveness and no conclusions can be drawn from those that did [24,29,38].

Many (20/24) studies reported on effectiveness in reducing mosquito densities. In terms of methods used to reduce vector density, insecticides and larvicides were used most frequently in high-income, urban settings (or experimental conditions that mimicked these settings). Larviciding was generally effective in experimental studies [27,28,33], although one study found no significant difference between treated and untreated sites [26]. Where larviciding was significantly more effective at reducing vector densities, drawbacks included labour requirements [29]. *Wolbachia* when used for population suppression, is potentially promising for reducing vector densities in highly urban areas [31]. Bti, as a target-specific bacterial larvicide, is potentially useful in densely populated urban areas [24–30] and may also overcome residents’ concerns about environmental effects or smell, which was an issue for some insecticide methods [34]. However, potential risks of Bti, including persistence and related environmental accumulation, could increase the risk of resistance.

Cost-effectiveness discussion was very limited, though engaging communities in breeding site reduction was routinely considered positively and appeared cost-effective (and increased sustainability) in Germany [24] and Uruguay [38].

Many studies identified community education and engagement [1], especially in densely-populated areas, as a relatively cheap, intuitive, and frequently used dengue control intervention. This was reflected by nine studies actively involving community members in delivery of interventions, primarily source reduction through removal or destruction of containers suitable for vector breeding [17,19,23–25,34,36,37,38]. Although most reported good community engagement and positive effects, one noted that households visited by public health staff actually contained more breeding sites than control households, possibly because of assuming they were sufficiently protected [37]. This has implications for policy and practice, indicating that community education and engagement is important, but must be ongoing and monitored/evaluated. While community engagement was clearly important, it was impossible to synthesise lessons on engagement approaches, quality, quantity, or relative contributions, as this was not clearly defined in studies.

### Limitations

Several limitations should be considered. We only included sources with an English abstract, so relevant documents in other languages may have been excluded. We may have missed documents that were only indexed in databases we did not include in our search. We did not appraise the quality of our sources, as this was a scoping review, intended to identify as broad and diverse a range of eligible documents as possible.

### Conclusions

Our main conclusion is that much more research should be conducted and published to strengthen the evidence on the effects of existing DENV vector control methods on dengue incidence/prevalence or mosquito vector densities in high-income, city settings. This is a significant evidence gap as DENV continues to increase its global reach.
